# Multiple constraints compromise decision-making about implantable medical devices for individual patients: qualitative interviews with physicians

**DOI:** 10.1186/s12911-017-0577-3

**Published:** 2017-12-22

**Authors:** Anna R. Gagliardi, Ariel Ducey, Pascale Lehoux, Thomas Turgeon, Jeremy Kolbunik, Sue Ross, Patricia Trbovich, Anthony Easty, Chaim Bell, David R. Urbach

**Affiliations:** 1 University Health Network, Toronto, Canada; 2 University of Calgary, Calgary, Canada; 3 University of Montreal, Montreal, Canada; 4 Concordia Hip and Knee Institute, Winnipeg, Canada; 5 Mount Sinai Hospital, Toronto, Canada; 6 University of Alberta, Edmonton, Canada; 7 University of Toronto, Toronto, Canada

**Keywords:** Medical devices, Decision-making, Influencing factors, Qualitative research

## Abstract

**Background:**

Little research has examined how physicians choose medical devices for treating individual patients to reveal if interventions are needed to support decision-making and reduce device-associated morbidity and mortality. This study explored factors that influence choice of implantable device from among available options.

**Methods:**

A descriptive qualitative approach was used. Physicians who implant orthopedic and cardiovascular devices were identified in publicly available directories and web sites. They were asked how they decided what device to use in a given patient, sources of information they consulted, and how patients were engaged in decision-making. Sampling was concurrent with data collection and analysis to achieve thematic saturation. Data were analyzed using constant comparative technique by all members of the research team.

**Results:**

Twenty-two physicians from five Canadian provinces (10 cardiovascular, 12 orthopedic; 8, 10 and 4 early, mid and late career, respectively) were interviewed. Responses did not differ by specialty, geographic region or career stage. Five major categories of themes emerged that all influence decision-making about a range of devices, and often compromise choice of the most suitable device for a given patient, potentially leading to sub-optimal clinical outcomes: lack of evidence on device performance, patient factors, physician factors, organizational and health system factors, and device and device market factors. In the absence of evidence from research or device registries, tacit knowledge from trusted colleagues and less-trusted industry representatives informed device choice. Patients were rarely engaged in decision-making. Physician preference for particular devices was a barrier to acquiring competency in devices potentially more suitable for patients. Access to suitable devices was further limited to the number of comparable devices on the market, local inventory and purchasing contract specifications.

**Conclusions:**

This study revealed that decision-making about devices is complex, cognitively challenging and constrained by several factors limiting access to and use of devices that could optimize patient outcomes. Further research is needed to assess the impact of these constraints on clinical outcomes, and develop interventions that optimize decision-making about device choice for treating given patients.

**Electronic supplementary material:**

The online version of this article (10.1186/s12911-017-0577-3) contains supplementary material, which is available to authorized users.

## Background

The increased availability and use of new and more complex medical devices has been referred to as an “explosion” [1]. Medical devices include a wide range of health or medical instruments essential for the prevention, diagnosis, cure or treatment of a disease or abnormal physical condition [1]. In the United States alone annual sales of medical devices were reported to increase from $85 billion in 2001 to $146 billion in 2009 [2]. While medical devices sustain life, and contribute to health and well-being, failures of implantable devices such as cardiovascular or joint implants have been associated with morbidity and even mortality [3, 4]. For example, analysis of Food and Drug Administration (FDA) device malfunction reports from 1990 to 2002 found the mean annual replacement rate was 20.7 per 1000 for implantable cardioverter defibrillators (ICDs) and 4.6 per 1000 for pacemakers, and 61 deaths (31 ICD, 30 pacemaker) were attributable to device malfunction (3). Among 30,002 devices approved by the FDA between 2005 and 2012, 249 were recalled, half during the first 2 years on the market [4]. Studies analyzing the quality of research for frequently emerging new versions of medical devices found they are marketed without the rigorous scientific evidence expected for drug approval and, thus, adverse medical device events may only emerge when they are used in patients [5, 6]. While registries can generate post-market evidence of device performance, they do not systematically and consistently capture data on every type of device, and are not widely available because they are costly to develop and maintain [2]. Given the potential risks associated with medical devices, and variable evidence of their safety and effectiveness, there is a need for greater insight on how physicians choose devices that optimize patient treatment and outcomes from among the available options.

Medical decisions are often informed by syntheses of explicit knowledge on the effectiveness and safety of a given medical innovation [7, 8]. However, multiple, often interacting factors can influence decision-making including attributes of the medical innovation itself, and the characteristics of patients, providers, organizations and health systems [9]. Thus decision-making is often complex and not strictly driven by the quantity and quality of evidence for a given treatment. Gabbay and le May studied treatment decision-making among primary care clinicians in two general practices in the United Kingdom and found that physicians rarely accessed and used explicit forms of evidence [10]. Instead they relied on “mindlines”, which they referred to as internalized guidelines. Mindlines integrated tacit knowledge, meaning non-codified internalized knowledge from their own experience, and from colleagues, opinion leaders, pharmaceutical representatives and patients, and were mediated by organizational demands and constraints. The concept of engaging patients in decision-making about their own care, which contributes to the formation and shaping of mindlines, has gained momentum as a health system priority world-wide because it improves patient outcomes and lowers costs [11].

As there is little data on the performance of many medical devices, empirical research is needed to examine the basis upon which physicians choose medical devices for individual patients, and understand if and how decision-making about medical devices could potentially be improved based on the mindlines concept by creating opportunities for tacit knowledge sharing among physicians, supporting patient engagement in decision-making and limiting organizational or external constraints on decision-making [12]. Such research may reveal the type of quality improvement efforts required to better support decision-making, and avoid or minimize adverse medical device events causing morbidity and mortality. The purpose of this study was to explore factors that influence individual physician decision-making about choice of implantable medical device from among options available to treat a given patient, and identify factors that may constrain choice and potentially compromise clinical outcomes, which should be targeted in the future through policies or behavioral interventions.

## Methods

### Approach

Given the lack of previous research on decision-making about medical devices, empirical description is needed to generate knowledge that could serve as the basis for future research. Thus a qualitative study design was used to thoroughly explore processes and determinants of decision-making [13]. More specifically, an approach called descriptive qualitative research was employed [14]. This method does not test or generate theory; instead it gathers straightforward accounts of views and experiences. Rigor and transferability were optimized using standard strategies that complied with the Consolidated Criteria for Reporting Qualitative Research [15, 16]. The University Health Network Research Ethics Board approved this study. Participants provided written informed consent prior to being interviewed. The research team had no relationship with any participants.

### Sampling and recruitment

Physicians were identified in publicly available certification agency directories, and hospital or university web sites, and invited to participate by regular or electronic mail. Purposive sampling was used to recruit physicians with a range of characteristics that could influence views and experiences including specialties that use implantable cardiovascular (cardiac or vascular surgeons, interventional cardiologists) and orthopedic devices (orthopedic surgeons), geographic region (different provinces in Canada) and years in practice (self-reported as early, mid and late career). A reminder was sent to non-respondents at two and 4 weeks. Qualitative research gathers in-depth data from a small number of participants. We anticipated interviewing 20 physicians, 10 for each of cardiovascular and orthopedic devices. In our experience of recruiting physicians for qualitative interviews, 5% to 10% of invited individuals agree to participate, therefore we over-sampled. Sampling was concurrent with data collection and analysis, and proceeded until no further unique themes emerged from successive interviews (saturation).

### Data collection

The principal investigator, a PhD-trained researcher with experience in the use of qualitative methods to explore factors that influence patient and provider behaviour, conducted telephone interviews of an average of 30 min. Participants were asked three questions about medical device decision-making: *How do you decide what type or model of device to use in a given patient*, *What sources of information do you consult*, and *How are patients engaged in decision-making*? Depending on responses, they were asked to comment on patient, physician, organizational or system factors that influenced decision-making. Interviews were conducted between April 8 and September 28, 2015, audio-recorded and transcribed.

### Data analysis

The principal investigator inductively identified and organized themes using Microsoft Word and Excel [13]. Transcripts were read to identify and define themes (first level coding). A codebook was developed to organize themes and sample quotes. Transcripts were re-reviewed (constant comparative technique) to assess whether and how to expand or merge themes (second level coding). Each of first and second level coding was independently reviewed by all members of the research team at two separate meetings. Saturation was determined by discussion and consensus among the research team at a third meeting. Apart from the principal investigator, the research team was comprised of eight investigators; five were investigators with expertise in social sciences, quality improvement, patient safety, human factors research, and health technology design, assessment and use, and three were clinician investigators with experience in surgery, implantable devices, and patient safety. Data were tabulated by theme and summarized. The summary was reviewed and discussed by the research team at a fourth 1-day meeting to interpret data.

## Results

### Participants and devices

Of 561 physicians invited to participate, 534 did not respond. Of 27 who consented, five could not be reached to schedule interviews, and interviews were conducted with 22 (Table 1). These included eight, ten and four early, mid and late career physicians, respectively from five different provinces. Ten interviews focused on cardiovascular, and 12 on orthopedic devices. Cardiovascular devices mentioned by participants included accessories (screws, leads), artificial hearts, cannulae, implantable cardiac defibrillators, pacemakers, stents and valves (tissue, mechanical). Orthopedic devices mentioned by participants included accessories (nails, screws, aiming devices), elbow prostheses, hip prostheses (hemi, total), locking plates, knee prostheses (unicompartmental, total), resurfacing caps or cups (hip, knee) and rods (femur, spine).

**Table 1:** Demographic characteristics of interview participants

Physician specialty	Self-reported career stage			Subtotal
	Early	Mid	Late	
Orthopedic surgeons	10OE-MB 11OE-MB 14OE-AB 15OE-NS 16OE-NS 17OE-NS	06OM-ON 08OM-MB 12OM-BC	03OL-ON 07OL-ON 09OL-MB	12
Cardiac or vascular surgeon, or cardiologists (general, electro-physiologists, interventional)	02 CE-ON 04 CE-ON	01CM-ON 05CM-ON 13CM-MB 19CM-ON 20CM-AB 21CM-ON 22CM-MB	18CL-ON	10
Subtotal	8	10	4	22

C cardiac, O orthopedic; E early career, M mid-career, L late career; two letter code for province: AB Alberta, BC British Columbia, MB Manitoba, NS Nova Scotia, ON Ontario

**Fig. 1:**
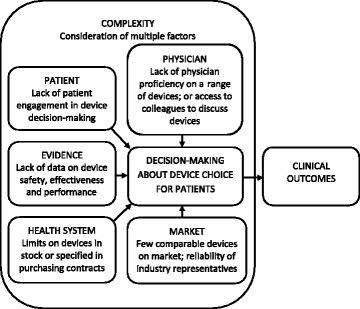
Factors constraining decision-making about choice of medical devices.

Additional file 1 presents exemplar quotes, which are discussed here to illustrate themes about factors said to influence decision-making about device choice. Responses did not differ by specialty, geographic region or career stage. Overall, five major categories of themes emerged, which all influence the choice of implantable devices to treat given patients, often functioning together. These factors often compromise choice of the most suitable device for a given patient, potentially leading to sub-optimal patient outcomes. Constraining factors are summarized in Fig. 1.

### Evidence on device performance

Participants described a lack of high-quality data from the medical literature on the safety and effectiveness of devices to inform decision-making. They noted that most studies were small cohorts with short follow-up time such that potentially adverse events associated with the device may not yet have occurred. Few participants said they had access to data on device performance from registries, and that even registry data was limited in scope and data quality. As a result, they said that devices could only be chosen based on “group consensus”. The evaluation of devices concurrent with real-time use in patients was referred to as a “beta test”, emphasizing that information about device performance or associated poor outcomes emerged over many years.

There is really nothing in the literature that is helpful on this. Everything we do in orthopedics is a beta test. You know how drugs go through phases of testing? There’s nothing equivalent to that in orthopedics. Someone just comes up with what they think is the latest and greatest idea and before long it’s a product and someone does a few [cases] and then it’s just on the market…. Maybe 10 years ago we started to see what are called locking plates and that’s where screws lock into the plate rather than just squeezing the plate against the bone….So people started putting them in left, right and centre, which caused all sorts of problems because we actually really didn’t understand what they were doing at the biology level. It took 3 or 4 or 5 years to try and figure that out and figure out the modes of failure and what you should do or shouldn’t do. So it’s just one big running beta test (06OM)

### Patient factors

Participants said that “best fit” for the patient was a predominant factor that influenced choice of device, referring to several patient characteristics including anatomy and pathology. Participants said that device characteristics and, hence, choice of device was less crucial in cardiovascular patients facing life-or-death situations, and among older patients for whom any orthopedic implant would likely last their lifetime. Thus decision-making was said to be on a “case by case” basis. Most participants believed that patients would not have sufficient knowledge to engage in decision-making about device choice and were thus involved only in informed consent about the device chosen for them.

### Physician factors

Participants varied in their stated predilection for experimentation with new devices. Most participants continued to use the same device on which they were trained, referring to themselves as “late adopters”, and suggesting that familiarity with the “nuances” of the device may be associated with optimal patient outcomes. Conversely, others referred to a “philosophy” of acquiring proficiency in a range of devices so that choice of device could be tailored to patient needs, thereby optimizing outcomes. Still others expressed interest in new devices as the basis for academic research, or because they were cutting-edge products.

Colleagues were the most frequently consulted source of knowledge when considering a new device, or to learn about others’ experiences with the same device after experiencing an adverse event. They included mentors, experts, and local, national or international colleagues. Interaction was often informal and occurred when needed. This was considered a quick and easy way to acquire trustworthy information. Interaction also took place on a more formal basis at various types of professional meetings.

### Organizational and health system factors

Choice of device was sometimes limited by what happened to be in stock in the hospital at the time of surgery. Other hospitals did not own or stock devices; instead physicians ordered devices from a “menu” and they were delivered to the operating room by industry representatives.

Participants were conscious that cost influenced access to devices from which they could choose. Costs were commonly managed through purchasing group contracts with preferred vendors. When multiple devices were appropriate for a given patient, participants said that they would “fulfill my contractual obligations”, referring to devices specified in group purchasing contracts. Differing views were articulated about the implication. Some noted that it was not always possible or necessary to use the “best of the best” or the “latest and greatest” in every patient. Others said that group purchasing contracts constrained choice, leading to poor patient outcomes, which would negate any costs savings.

Sometimes the implant you put in is not what you think is the best for the patient because that’s the only thing available through the buying group (07OL)

One of the biggest deciding factors will be cost and not necessarily surgeon comfort, patient anatomy and track record of implant. We’ve had experience that if you force surgeons to change implants based on a contract that your complication rate goes up for a while. That is problematic when it occurs. So it makes good business sense until you actually go and look at your revision costs over the next months to 2 years and then, all of a sudden, all of your cost-savings went into pain and suffering of patients and their subsequent care (08OM)

### Device and device market factors

Decision-making was sometimes limited to one or two models available on the market, for example, implantable ventricular assist devices. Similarly, certain types of device components were considered comparable, for example, “a screw is a screw”. Participants referred to the comparative features or merits of devices, noting that most devices for a particular clinical indication marketed by different companies were very similar, and they would switch to a newer device only if it offered advantages in terms of “ease of implantation or safety”.

Apart from colleagues, participants often relied on industry representatives to provide information about devices and who uses the device. However, a few participants noted that some representatives did not consistently share information about device warnings, requiring physicians to double-check information provided by representatives with colleagues or switch to other products upon experiencing an adverse medical device event.

For example, there was a problem with a type of wire that we use in coronaries where the manufacturer knew there was a problem and left them on the shelves. So I basically stopped using any of their stuff (21CM)

### Multiple factors

Some participants said that multiple sources of data were considered including medical literature, Internet, registries, colleagues, professional meetings and industry representatives along with multiple factors such as patient characteristics, and device cost, availability and familiarity, underscoring the complexity of decision-making about choice of device.

## Discussion

Physicians who use a range of implantable cardiovascular and orthopedic devices that were interviewed said, in the absence of explicit evidence from scientific research or device registries, they gathered and integrated tacit knowledge from various sources to choose the best possible medical device for addressing patient-specific needs. They most frequently consulted with trusted colleagues, often on an informal, as-needed basis. They also consulted with industry representatives, although this information was viewed by participants as less trust-worthy. Patients were rarely engaged in decision-making. Physician preference for particular devices was viewed as a potential barrier to acquiring competency in a range of devices that could be more suitable for patients. Access to devices considered suitable for patients was further limited to the number of comparable devices on the market, local inventory and devices specified in purchasing contracts. Thus interacting evidence, patient, physician, organization, health system and device market factors influenced decision-making. Overall, this study revealed the complexity of medical decision-making to choose devices, which is constrained by the cognitive challenge of acquiring, verifying and integrating knowledge from various sources and then tailoring it for individual patients, and further constrained by several factors limiting access to and use of devices that could optimize patient outcomes.

Our findings are similar to those of Pope [17] who interviewed 34 general surgeons, gynecologists and urologists about treating women with urinary incontinence and found that surgical decisions and actions were contingent upon patient factors that were readily apparent (anatomy, age, clinical history, comorbidity), surgeon factors that were complex and less clear (personal preferences, technical options available) and external factors that were the least obvious (availability of equipment, resources, operating room staff, time, broader environment). Surgeons needed to constantly adjust and adapt to these contingencies. Our findings are also similar in many respects to research that examined decision-making in contexts other than surgery. Wadmann studied prescribing among general practitioners in Denmark and found that they acquired information from professional societies, colleagues and medical opinion leaders [18]. The physicians in the Wadmann study said that it was challenging to acquire and organize this information, and that visits from sales representatives were helpful because they offered decision support tools such as summaries and flow charts. Gold conducted interviews with oncologists in the United States regarding decision-making about the use of accelerated partial breast radiotherapy [19]. Some physicians said they were influenced to use the technology because it was beneficial for patients; some said that it offered financial advantages through referrals or compensation, improved one’s reputation as an early adopter, or they were emulating other physicians; and others were less inclined to use it because they were reluctant to learn something new, or had concerns about malpractice.

Strengths of this study included the use of purposive sampling to recruit participants who varied by characteristics that may have influenced their views or experiences including specialty, geographic setting and years in practice, and rigorous methods for data collection and analysis. More importantly, we sampled to thematic saturation, in other words, to the point where no further unique information emerged from successive interviews which, in qualitative research, signals that recruitment is sufficient. Still, the interpretation and application of these findings may be limited by several issues. Participants described factors that influenced choice of device in general; they may have responded differently if asked about particular types of orthopedic or cardiovascular devices. A small proportion of those invited participated in interviews thus their representativeness of the larger population of physicians who implant orthopedic and cardiovascular devices is unclear. Participants were sampled from Canadian hospitals so the findings may not be transferrable to other settings. However, the devices used by our participants are also used by physicians worldwide, therefore the views and experiences reported here, which were similar to those that emerged from other studies of decision-making [10, 17–19], may be broadly relevant.

Several implications are raised by this study, which suggests avenues for ongoing research. Due to the nature of device development and marketing, it is unlikely that licensing criteria and processes will change [1]. It is probable that devices will continue to be tested in real-time once they are marketed. The concept of mindlines [10, 12] and the findings of our study suggest that medical device decision-making could be supported by enabling the sharing of tacit knowledge among colleagues. Communities of practice refer to professional networks that form organically to share information, however, it is recognized that such networks can be more successful if they are stewarded and provided with support to facilitate knowledge sharing [20]. Such networks could also support surgical mentoring for continuing professional development among colleagues with competence in different types of devices. Given the priority of health systems on patient engagement and the apparent lack of patient engagement in medical device decision-making, ongoing research is needed to understand how to best engage patients in discussions and decisions about medical devices, and also how to prompt and support physicians to engage their patients in such discussions.

Industry representatives emerged as another source of device information. Research has found that physicians’ clinical decisions and practice patterns can be influenced by industry relationships [21, 22]. We have little understanding of the role or influence of device industry representatives on the selection and use of devices by individual physicians. Nursing and surgeon professional associations have issued statements regarding the credentialing and appropriate role of medical device representatives [23, 24]. Yet a survey of senior nurses in charge of 79 gynecology operating theatres in the United Kingdom found that 82% had no guidelines for representative presence in the operating room, and 42% obtained patient consent for such visits [25]. Further research is needed to understand these relationships, and their impact on physician decision-making and on patient outcomes.

This study revealed that some physicians were required to use devices stipulated in purchasing agreements even if they did not feel entirely competent with those devices or thought they were not an ideal choice for a given patient. Such hospital policies seek to optimize the efficiency of supply management, standardize surgery and reduce costs through “just-in-time” delivery of medical devices by vendor representatives [26]. However, a survey of orthopedic surgeons in Pennsylvania found that they were less aligned with hospital purchasing managers who promoted cost containment, and more aligned with device representatives with whom they had long-standing relationships, and who provided them with financial or service benefits [27]. Further research is needed to evaluate whether the device restrictions imposed by purchasing groups may be associated with poor patient outcomes.

## Conclusions

This study explored factors that influence decision-making for treating patients with a range of implantable devices. Twenty-two physicians from five Canadian provinces (10 cardiovascular, 12 orthopedic, 8, 10 and 4 early, mid and late career, respectively) said that, in the absence of evidence from research or device registries, tacit knowledge from trusted colleagues and less-trusted industry representatives informed device choice. Patients were rarely engaged in decision-making. Decision-making was further influenced by patient, physician, organizational, system, device and device market factors. Physician preference for particular devices was a barrier to acquiring competency in devices potentially more suitable for patients. Access to suitable devices was further limited to the number of comparable devices on the market, local inventory and purchasing contract specifications. Thus, decision-making about devices is complex, cognitively challenging and constrained by several factors limiting access to and use of devices that could optimize clinical outcomes. Further research is needed to assess the impact of these constraints on clinical outcomes, and develop interventions that optimize decision-making about device choice for treating given patients.

## Additional file

Additional file 1: Factors that influence choice of implantable medical device (DOCX 22 kb)

## Abbreviations

FDA: Food and Drug Administration.
ICD: Implantable cardioverter defibrillator
