# Supplementary material for: Multiple constraints compromise decision-making about implantable medical devices for individual patients: qualitative interviews with physicians
Source: BMC Med Inform Decis Mak. 2017 Dec 22;17:178. doi: 10.1186/s12911-017-0577-3 (PMC5741896; doi:10.1186/s12911-017-0577-3)
Supplement: Additional file 1: — Factors that influence choice of implantable medical device (DOCX 22 kb) [file 12911_2017_577_MOESM1_ESM.docx]

Additional file 1. Factors that influence choice of implantable medical device

| **Theme** | **Cardiovascular devices** | **Orthopedic devices** |
| --- | --- | --- |
| **EVIDENCE** |  |  |
| Evidence on safety and effectiveness in the medical literature (or lack of) | The evidence-base is usually not great. But there’s some data that allows us to feel ethically and medically responsible to use these devices (02CE)  There aren’t many large randomized trials for decision-making. Things that come to the literature are relatively small series or cohort studies. So it’s often through group consensus (22CM) | A lot of the things that we do, there’s not too many things, a case study is written but that’s not really good science, it’s just a report, there’s no trials about some of these things (10OE)  The trouble with the medical literature is you’re looking at data that gives you a two-year outcome and that’s good data if you can get it. You’re looking at 10% of the published literature that gives you two-year outcomes on implants that could possibly show maybe even early failure. When we’re talking with patients we’re talking 10 to 15 years down the road, how well do these implants work? Decision-making on implant selection can’t be from the medical literature and it’s not appropriate to do so because you’re narrowing your focus on only two-year outcome data (14OE) |
| Data on device performance from registries (or lack of) | We participate in a registry for vascular surgery and when we do our follow-up of our endovascular repairs we track these complications. We’re the first Canadian site to participate in this database that involves over 300 U.S. centres. We pay for this and we contribute data and are able to see our results in real-time (19CM) | Registry data may not be the greatest data in terms of how clean the actual data is but at least you get a better sense of what 15 to 20 years down the line how implants are functioning. Registry data has the longest term outcome. There are certain registries out there that guide us as to which implants are good and which are not, which have more longevity. Australia has an excellent registry and Denmark has a registry. U.S. and Canada are quite far behind (14OE) |
| **PATIENT** |  |  |
| Physiological characteristics | It all depends on what has the best fit for the patient. For example, you have a choice of either rings or bands. I use a ring when I have pathology that involves the entire valve. If it just involves the posterior aspect of a particular valve then I just use a band. So I use devices based on patient characteristics, patient problem and also what is best for the patient (01CM)  That’s based on a review of the patient’s anatomy (19CM)  The overriding factor is what I think will be the best fit for the patient (21CM) | I try to look at the patient’s issues and find the best solution available (10OE)  It’s really on a case by case basis (11OE) |
| Age | --- | If you’re talking about an older patient whichever device you put in will probably last them (09OL) |
| Medical necessity | These patients are facing life or death situations and the device is really their only chance at life so it doesn’t really affect our use of the device (05CM) | --- |
| Engagement in decision-making | I’m not so sure that they’re gonna have the insight to be informed enough about which device is gonna be better for them (04CE)  We do involve patients though probably not separate from the consenting process. I’m not quite sure at what level and what kind of detail a patient would understand (13CM) | I think that the general population to be quite frank is not smart enough to engage in that discussion. Physicians quite frankly don’t have the time to educate people. Even in the basics that they would have to know. That sounds really paternalistic and I should probably apologize for that but I just can’t see that as being workable. That that is one of those areas where people have to make decisions on behalf of their patients and the patients have to trust their providers to make those decisions (06OM)  If you’re just using the same good old standard , and most cases would be in that category, I don’t think that it warrants much discussion (11OE) |
| **PHYSICIAN** |  |  |
| Physician preference based on familiarity and training | A large part of it is based on what I was exposed to as a trainee, because what you implant as a trainee, that’s comfortable and the nuances of the device are known to you (20CM) | I probably would be called a late adopter. The biggest change I made recently was to start doing reverse arthroplasty. It was introduced in Europe twenty years ago and in North America ten years ago but I didn’t start doing it until the manufacturer developed systems where, if it failed, you could convert it to the conventional arthroplasty (03OL)  If I’ve never used that stem before, even the track record looks really good on that implant, because I’ve never used it that patient is now part of my learning curve (08OM)  When I offer surgery I already have in mind which implant I’m gonna use and it’s the one I’m comfortable with (11OE)  Based on experience with the one you are trained on in residency or fellowship, then you usually continue on the same pathway (16OE) |
| Physician preference based on philosophy or interest in innovation | We also have an academic interest in introducing new devices. New technology doesn’t have clinical track records. We’re the ones generating the data to achieve approval (05CM) | We’re like magpies in orthopedics. We like bright shiny new toys (06OM)  Some people treat everything with one system and some other people treat tailored to the patient. It depends on your philosophy and your training. I’m one of those people that tailor it so I try to look at the patient’s issues and find the best solution available (10OE)  Some people may feel they’re only comfortable sticking with one. But being an arthroplastic surgeon is complex. You need a variety, and there are benefits and down sides to every single implant in terms of correcting for deformities, problems, variations on normal anatomy. In order to give the best outcome for patients, the one or two implants that you’re comfortable with may not correct those issues and that’s why I feel the need to use a wide variety of implants (14OE) |
| Opinions and experiences of colleagues (informal) | We reach out to colleagues across the country or even the world (02CE)  If there’s device that’s completely foreign to us and we know that a colleague across the country or even North America has had some experience it wouldn’t be unusual for us to call them and say you know I’ve been approached to trial this device, what do you think about it, what are the advantages, the disadvantages? (05CM)  Usually we hear about new devices from either your colleagues or the device reps. If it’s my colleague, you trust them to give you all the details of what to do, what not to do and what’s an issue (20CM) | It’s the people that I respect in terms of my mentors and other people that have done this a long time, and my own contemporary colleagues as well (10OE)  I email my previous mentors and colleagues a lot to see what they think (11OE)  I speak with my colleagues who have used the device. There’s 40 orthopedic surgeons within a 40 kilometer radius so I’ll just call some of them and ask. I will occasionally speak to someone back where I initially trained (12OM)  I might talk to colleagues. Start with local and then move on to elsewhere. If you’re thinking about a device and know that you’re gonna see someone at a meeting you might reserve your discussion and questions for then. But it’s really easy to reach out to people via email and a phone call (15OE) |
| Opinions and experiences of colleagues (meetings) | At scientific conferences well known experts who use a device, they come and present the results of their observations and experiences, bad and good, and share it (18CL)  What happens is things get approved most easily in Europe, so the Europeans get to play with things a year before North America would have access. Usually the Europeans have been using something for a while before we get to touch it. So I’ll speak to somebody in a meeting and ask them what they think about it (21CM) | Like any specialty we have lots of meetings. That’s probably one of the primary places you begin to learn about things. We often have case-oriented discussions or panel discussions (03OL)  I go to a lot of orthopedic meetings where implants are discussed and you do get a lot of reports that some instrument companies have had recalls or some kind of issue has come up with their implant (07OL)  We have a <regional> orthopedic meeting once every two months and if anything comes up that would be mentioned there (09OL) |
| **ORGANIZATION/HEALTH SYSTEM** | |  |
| Devices in stock | The device selection itself, that’s limited by what we have available (22CM) | Sometimes we just don’t stock things. We might not carry it in our regular inventory. It doesn’t make sense to carry it if you’re only doing one or two a year (06OM)  The hospital doesn’t own the equipment anymore. Everything comes in on consignment. We figure out the size or you know length or whatever it is we need and then he picks it off his selection. It’s like looking at a menu. The hospital is charged appropriately. They actually carry in the instruments to do the operation because the hospital doesn’t own them (07OL) |
| Devices approved by purchasing agreements with specific vendors | There are contractual obligations that would make me try one device more than another. In cases where I can use multiple devices then I would try and fulfill my contractual obligations (13CM) | 90% of the implants that we put in are supposed to be <company name> (17OE)  Sometimes the implant you put in is not what you think is the best for the patient because that’s the only thing available through the buying group (07OL) |
| Cost of devices | Finance affects your decision-making for sure. I just can’t use the latest and greatest of everything. That’s just not possible. But you don’t necessarily need to use the latest and greatest for everything. Some [device] features are helpful and others are less helpful. The value of it depends on the benefit to the patient (21CM) | Surgeons and physicians need to be conscientious about the finances in our health care, you can’t be implanting the best of the best in every single person. We have to be selective to some degree (14OE) |
| **DEVICE/DEVICE MARKET** | |  |
| Comparative advantage or benefit | They’re all very similar and there’s no real significant advantage between one and another (01CM)  New is not necessarily better. There are some devices where I’m not switching because it’s doing everything I need it to do, and other situations where an iteration of a device provides very helpful advantages in terms of ease of implantation or safety (21CM) | We don’t really know a whole lot about how much of a difference there is between implant X versus implant Y (17OE)  I don’t try very many new things unless there’s no alternative or I’m convinced that they’re better than the existing device (03OL) |
| Devices on the market are few or standardized | In the world of implantable ventricular assist devices, there are only two available devices on the market now that are being used predominately around the world and in our institution (04CE) | There are reports about certain devices in the literature. A lot of those reports are older. For pedicle screws the literature would be from the 1980s and early 1990s. We don’t really have a lot of data. It’s not really a hard thing to perfect. A screw is a screw (17OE) |
| Vendor representatives | You ask a rep to come and give a presentation to the entire division about this particular product (01CM)  The company reps are the ones that provide us with the data. The company rep would say here’s device A and here is the published data on device A. They will tell us here’s a cohort study of 300 patients who had this valve and everything went well for the duration of the study. Would you be interested in using this and adding to the clinical experience and performing your own evaluation (05CM)  If we ever got into a situation where I felt that they were putting patients at risk I would just go crazy on them. For example, there was a problem with a type of wire that we use in coronaries where the manufacturer knew there was a problem and left them on the shelves. So I basically stopped using any of their stuff (21CM) | There is a lot of information directly from the manufacturers (07OL)  There are a number of other companies we use where the representation falls well below the standard and it’s well-known. In relationships like that your guard is up, you’re always double-checking everything, you’re always verifying with other colleagues that have used that system to make sure that you’re doing the best you can for the patient (10OE)  They’re always available if we want to meet to discuss a product. They’ve been good at saying some people don’t use this, this other one is more simple, so they do keep us posted on what other people use more often (11OE) |
| **MULTIPLE** |  |  |
| Combination of patient, physician and external factors | It depends on the patient’s age, their life expectancy, what options we have available at that point in time – we have certain devices that are available in house, what team members we have to actually implant the device, and the referral centre – what capability they have. It would depend on what we think the progression of care will be in terms of do we use a temporary device and which temporary device do we use, or whether they are appropriate for definitive therapy which would be transplantation. All those factors go into the equation (22CM) | There’s a bunch of factors. There’s a whole pile of companies that make similar devices. First of all, what’s the best device for the patient? If there’s multiple choices of a similar device then it comes down to cost, accessibility, familiarity for the nursing staff and myself. If it comes down to equality then I’ll just use what we have for ease of use (12OM) |
| Combination of sources of information | Every year at the major meetings, there’s publication on certain longevity results, the device reps are pretty quick to share their good results with you. Having access to that or reading an article dictates a lot of what we do (20CM)  From the literature, from the vendor, from meetings, from colleagues, from the Internet, from opinion leaders (21CM) | First of all you look at the registry data and see what’s out there and compare it to what options I have available. The second step is my experience with the actual implants themselves. Then the third step comes down to support from the companies in terms of being present if issues do arise (14OE) |
